# Static magnetic field inhibits epithelial mesenchymal transition and metastasis of glioma

**DOI:** 10.1038/s41598-025-96047-x

**Published:** 2025-04-11

**Authors:** Ziyu Sun, Wenxuan Zhao, Xifeng Fei, Bao He, Lei Shi, Zhen Zhang, Shizhong Cai

**Affiliations:** 1 Department of Neurosurgery, Gusu School, Nanjing Medical University, The First People’s Hospital of Kunshan, Suzhou, People’s Republic of China; 2 Department of Neurosurgery, Suzhou Kowloon Hospital, Shanghai Jiaotong University School of Medicine, Suzhou, People’s Republic of China; 3 Department of Radiology, Affiliated Kunshan Hospital of Jiangsu University, China Medical University, Gusu School Nanjing Medical University, Suzhou, People’s Republic of China; 4 Department of Child and Adolescent Healthcare, Children’s Hospital of Soochow University, Suzhou, Jiangsu People’s Republic of China

**Keywords:** Glioma, Magnetic field, Epithelial-mesenchymal transition, TGF-β1, Cancer therapy, CNS cancer, Metastasis, Tumour biomarkers

## Abstract

Gliomas exhibit suboptimal responses to conventional treatments, with tumor cell migration remaining a significant challenge in therapy. Epithelial-mesenchymal transition (EMT) is crucial for glioma cell invasion, and transforming growth factor β1(TGF-β1) is a key factor promoting proliferation, migration, and EMT in glioblastoma (GBM). Although magnetic fields are widely used in the diagnosis and treatment of various diseases, their effects on EMT in glioma cells remain unclear. In this study, we investigated whether a static magnetic field (SMF) could inhibit EMT and metastasis in glioma cells. Cellular functional assays using the U251 and U87 glioma cell lines were performed to investigate their functional and phenotypic changes. Results showed that TGF-β1 treatment increased the invasion and migration capabilities of glioma cells, while simultaneously reducing apoptosis. However, when SMF was combined with TGF-β1 treatment, a significant reduction in cell migration and invasion was observed, along with an increase in apoptosis. Additionally, this combination treatment significantly decreased the protein expression of mesenchymal markers N-cadherin and β-catenin, as well as reduced the levels of the matrix metalloproteinase (MMP)-2. Collectively, these findings suggest that SMFs may attenuate glioma cell metastasis by inhibiting EMT. Therefore, SMFs could represent a promising therapeutic strategy for diminishing glioma metastasis.

## Introduction

The incidence of central nervous system (CNS) tumors also varies by region. For instance, Europe has the highest incidence rates, with approximately 6.59 per 100,000 population, while in the United States, the rate is around 5.74 per 100,000. In contrast, Asia has the lowest rates, below 3 per 100,000^1^. Gliomas are malignant primary brain tumors believed to originate from neural stem cells or progenitor cells carrying tumor-initiating mutations^2^. These tumors are characterized by rapid proliferation, invasiveness, and poor prognosis, with a median survival period of approximately 14.6 months. The five-year survival rate for glioblastoma (GBM) is 5.1%^3^.

Current treatment for glioblastoma is a combination of surgery, radiation therapy, and chemotherapy. However, traditional treatments have proven ineffective in preventing tumor recurrence and metastasis. Recent studies suggest that epithelial-mesenchymal transition (EMT) may play a crucial role in tumor invasion and drug resistance. EMT is a reversible process^4^; its characteristics include loss of epithelial cell polarity, reduced intercellular adhesion, and enhanced cell migration capability. EMT has been proven to play a critical role in embryonic development, but its involvement in tumor metastasis in vivo remains controversial^5,6^. For example, during the formation of renal organs, the mesenchyme surrounding the ureteric bud develops into renal epithelium through EMT, which is then followed by the mesenchymal-epithelial transition (MET) process^7^. Similarly, EMT can facilitate the metastasis of tumor cells. Cancer cells undergo EMT, transitioning from an epithelial to a mesenchymal-like state, acquiring migratory and invasive capabilities to detach from the primary tumor site and migrate to distant locations. Upon reaching a new site, they undergo mesenchymal-epithelial transition (MET) to revert to an epithelial-like state, thereby promoting tumor growth at the metastatic site^8,9^. It is believed that cancer cells undergo EMT under the influence of various extracellular signals in the tumor microenvironment^10^. Major pathways involved include TGF-β, Wnt, Notch, and Hedgehog signaling pathways, all of which are associated with the process of EMT. Among these, the TGF-β pathway is likely the main inducer of EMT^11^. Additionally, cells exhibiting EMT characteristics typically degrade and invade the extracellular matrix by expressing matrix metalloproteinases (MMPs)^12^. The existence of EMT in glioblastoma remains controversial^13^, but the biological process of EMT is significantly associated with the prognosis of glioma patients^14^, indicating a close relationship between EMT progression and poor prognosis in glioblastoma^15^.

All organisms are daily exposed to magnetic fields (MF), which has increased concerns about their potential effects on human health. Due to the wide spectrum of frequencies, amplitudes, and intensities of magnetic fields, their direct biological targets are not yet fully understood, and their biological effects are diverse^16^. There is evidence suggesting that long-term exposure to MF may increase cancer incidence. In 2002, the International Agency for Research on Cancer (IARC) of the World Health Organization (WHO) classified static and extremely low-frequency (ELF) magnetic fields (300 kHz-300 GHz) as possible human carcinogens. However, certain specific intensities of magnetic fields have shown inhibitory effects on various cancers such as lung cancer^17^ and breast cancer^18^. The exact mechanism by which static magnetic fields affect tumors is still not fully understood. One proposed mechanism is that magnetic field induce reactive oxygen species (ROS)-mediated DNA damage, leading to cell apoptosis and ferroptosis^17,19^. Another hypothesis is that static magnetic fields inhibit tumor growth by reducing blood flow within tumor vasculature^20^ and suppressing angiogenesis^21^. In glioma, cell viability decreases significantly, possibly due to reduced expression of cyclin-dependent kinase 1(CDK1) protein, rather than apoptosis^22^. In our previous research, it has been confirmed that a static magnetic field of 1000Gs ± 100Gs has an inhibitory effect on glioma cells. However, the influence of static magnetic fields on the EMT process in gliomas is largely unknown.

This study aims to investigate whether static magnetic fields can inhibit EMT in gliomas. Our findings demonstrate that glioma cells, induced by TGF-β1, exhibit significant EMT characteristics, and when exposed to a static magnetic field (1000Gs ± 100 Gs), their migration and invasion abilities are markedly reduced. Moreover, apoptosis is increased, accompanied by a decrease in mesenchymal markers, including N-cadherin, β-catenin, and matrix metalloproteinase (MMP)-2. These results suggest novel therapeutic strategies for glioma treatment.

## Materials and methods

### Cell lines and cell culture

The human glioblastoma cell lines U87 and U251 were purchased from the Cell Bank of the Chinese Academy of Sciences (Shanghai, China) and cultured in high-glucose DMEM (Gibco/Biosharp), supplemented with 10% fetal bovine serum (FBS) (Gibco) and 1% penicillin-streptomycin (PS) (100 U/ml penicillin and 100 mg/ml streptomycin). GBM cells were maintained at 37 °C in a humidified atmosphere with 5% CO_2_, with medium changed every 2–3 days. For treatments, cells were exposed to 10 ng/ml TGF-β1 (PeproTech).

### Cell cloning and colony formation assay

U87 (300 cells/well) and U251 (400 cells/well) cells were seeded into six-well plates. After cell adherence, the experimental groups were treated with 10 ng/ml TGF-β1. Cells were allowed to grow for two weeks, then fixed with 4% paraformaldehyde (Suzhou Qiangsheng), stained with 2.5% crystal violet solution (Solarbio) for 30 min, washed, photographed, and counted.

### EdU proliferation assay

Cell proliferation was assessed using an EdU kit (Beyotime). Cells were seeded in 96-well plates at 3 × 10^3 cells/well (U87) and 4 × 10^3 cells/well (U251) and cultured for 72 h. The cells were then treated overnight with complete medium containing 10 µM EdU labeling reagent. The following day, cells were fixed with 0.5 ml of 4% paraformaldehyde (Suzhou Qiangsheng) for 30 min at room temperature, permeabilized with 0.5 ml of permeabilization buffer (Beyotime P0097) for 10–15 min at room temperature, and stained with Apollo staining solution and Hoechst 33,342 for 30 min each. Images were captured using a fluorescence microscope (Olympus IX73). The proliferation index was calculated as the percentage of EdU-positive cells relative to total cell count.

### Apoptosis assay

Cells were seeded at 3 × 10^4 cells/well (U87) and 4 × 10^4 cells/well (U251) in 12-well plates and treated with either a static magnetic field or TGF-β1. After 72 h, cells were collected and apoptosis was detected using an apoptosis assay kit (KeyGEN BioTECH), followed by flow cytometric analysis (BD FACS-Canto II) according to the manufacturer’s instructions.

### Migration and invasion assays

For the migration assay, cells were starved for 24 h, detached with trypsin, and resuspended in serum-free medium to a concentration of 4 × 10^4 cells/ml (U87) and 5 × 10^4 cells/ml (U251). A total of 200 µl of cell suspension was added to the upper chamber of a 24-well plate, while the lower chamber contained 500 µl of complete medium containing 15% FBS, with or without 10 ng/ml TGF-β1. For the invasion assay, the upper chamber was coated with matrix gel, diluted 1:10 in serum-free DMEM, pre-cooled at 4 °C and kept on ice. Sixty microliters of diluted matrix gel was added to each insert, ensuring smooth spreading without air bubbles. Plates were incubated in a cell culture incubator at 4 °C for 3 h until the matrix gel solidified completely. Cells were then seeded and incubated under standard conditions for 72 h. After fixation, cells were stained with crystal violet dye, and the final counting was performed after imaging.

### Western blot

Cells were lysed using cell lysis buffer (Jiangsu Cowin Biotech) and protein concentrations were determined using a BCA protein assay kit (Jiangsu Cowin Biotech). Equal amounts of protein were separated by SDS-PAGE and transferred to PVDF membranes, which were then blocked in 10% skim milk prepared in advance and shaken at room temperature for 1 h. PVDF membranes were incubated overnight at 4 °C with primary antibodies: mouse anti-GAPDH (1:2000; ABclonal), N-cadherin (1:5000; Proteintech), β-catenin (1:5000; Proteintech), and rabbit anti-MMP-2 (1:1000; Beyotime). The following day, membranes were shaken at room temperature and washed three times with TBST for ten minutes each. After washing, membranes were incubated at room temperature for 1 h with corresponding secondary antibodies: goat anti-mouse IgG (Beyotime) and goat anti-rabbit IgG (Beyotime). Subsequently, protein bands were visualized using chemiluminescence and exposed with an electronic imager (TOUCH IMAGER).

### Statistical analysis

Data were processed using ImageJ, FlowJo, and Photoshop 2020 software. All data were analyzed with GraphPad Prism version 10 and presented as means ± SD. Two-way ANOVA was used for statistical comparison and to make two-group comparison. *P* < 0.05 is considered statistically significant.

## Results

### TGF-β1 promotes migration and invasion of glioma cells

**Fig. 1:**
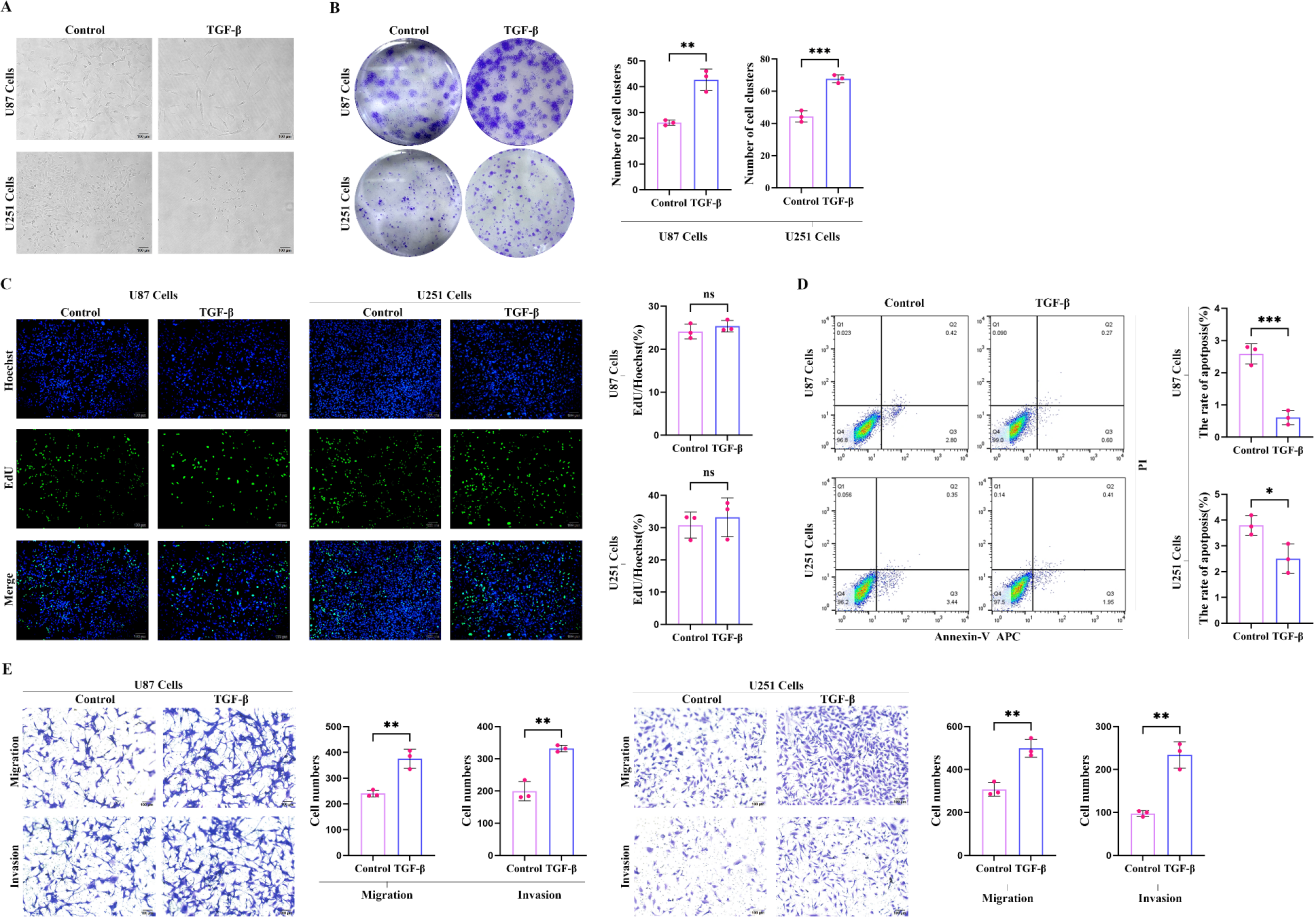
TGF-β promotes the proliferation, migration, and invasion of glioma cells. (**A**) Morphological changes in U87 and U251 cells after TGF-β1 treatment observed under an inverted fluorescence microscope at 10× magnification; (**B**,**C**) The effect of TGF-β1 on the proliferation ability of U87 and U251 cells; (**D**) A significant decrease in the number of apoptotic cells under TGF-β1 intervention compared to the control group; (**E**) In the Transwell assay, cell migration and invasion were assessed and cell counts from different groups were analyzed by t-test. Following 72 h of TGF-β1 treatment, the migration and invasion ability of GBM cells were notably increased. ns: *p* > 0.05; *p < 0.05; **p < 0.01; ***p < 0.001; ****p < 0.0001.

In many cell types, TGF-β1 is known to inhibit cell proliferation, particularly in epithelial and lymphocytic cells. However, in certain tumor cell types, TGF-β1 paradoxically promote invasion and migration. To investigate the effect of TGF-β1 on glioblastoma cells, U87 and U251 cells were treated with 10 ng/ml of TGF-β1 for 72 h. As shown in (Fig. 1A), TGF-β1-treated cells exhibited morphological changes, adopting a spindle-like shape resembling mesenchymal cells. In a cloning assay, it was confirmed that TGF-β1 may have a proliferative effect on U87 and U251 cells (Fig. 1B). To further validate the effect of TGF-β1 on cell proliferation, an EdU assay was employed. As shown in (Fig. 1C), after 72 h of TGF-β1 treatment, inverted fluorescence microscopy revealed a slight increase in proliferation compared to the blank control group. However, this increase was not statistically significant, indicating that TGF-β1’s main effect on glioma cells does not involve significant enhancement of their proliferative capacity.

Interestingly, apoptosis was significantly reduced in TGF-β1-treated U87 and U251 cells (Fig. 1D). To investigate whether TGF-β1 also affects cell migration and invasion, Transwell assays were conducted using U87 and U251 cells treated with TGF-β1 for 72 h. Compared to the control group, TGF-β1-treated U87 and U251 cells demonstrated significantly enhanced migration and invasion abilities (Fig. 1E). Thus, these findings suggest that TGF-β1 promotes migration and invasion of glioblastoma cells.

### Magnetic field inhibits glioblastoma cells treated with TGF-β1

**Fig. 2:**
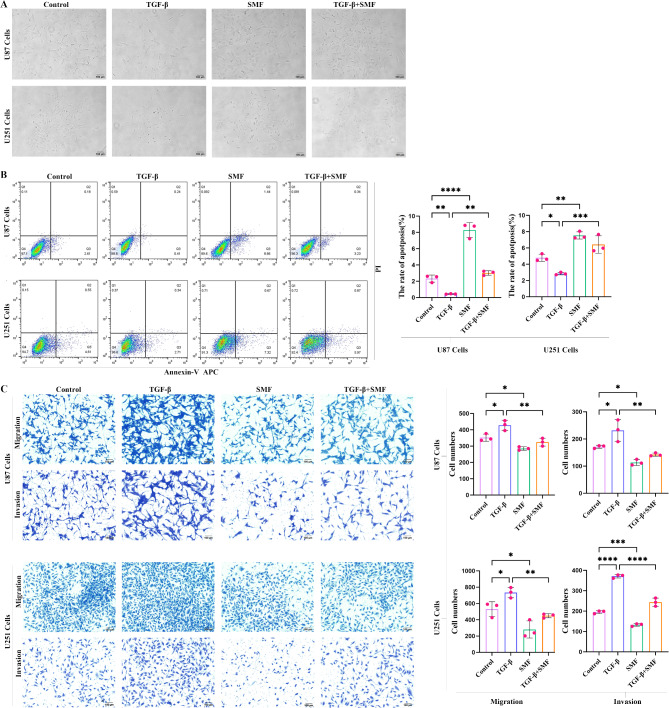
Static magnetic field inhibits TGF-β1-treated glioma cells. (**A**) Cellular morphological changes in U87 and U251 cells following treatment with TGF-β1, SMF, or the combination of both, observed under a 10× magnification using an inverted fluorescence microscope. (**B**) The static magnetic field increases cell apoptosis; after 72 h of TGF-β1 treatment, apoptosis decreases, but the combined treatment increases apoptosis. (**C**) The static magnetic field inhibits cell migration and invasion, whereas TGF-β1 enhances cell proliferation and invasion; combined treatment reduces cell migration and invasion. ns: *p* > 0.05; *p < 0.05; **p < 0.01; ***p < 0.001; ****p < 0.0001.

To investigate whether static magnetic fields (SMF) can modulate the cellular effects of TGF-β1, we treated U87 and U251 cells for 72 h with TGF-β1, SMF, or their combination. While SMF alone had minimal impact on cell morphology, it successfully reversed the morphological changes induced by TGF-β1, with this reversal being particularly evident in U87 cells (Fig. 2A). In addition, we employed Annexin V/PI dual staining to assess its impact on glioblastoma cell viability. The results showed that the static magnetic field increased the proportion of apoptotic cells in both U87 and U251 cells lines. After 72 h of TGF-β1 treatment, apoptosis levels decreased; however, when TGF-β1 treatment was combined with the static magnetic field, the proportion of apoptotic cells increased again (Fig. 2B). Furthermore, Transwell assays confirmed whether the static magnetic field could suppress the migration and invasion capabilities of glioblastoma cells (Fig. 2C). Compared to the control group, the magnetic field inhibited the migration and invasion of both U87 and U251 cells and reversed the migration-promoting effect of TGF-β1.

### The static magnetic field modulates the expression of EMT-related genes in glioblastoma cells

**Fig. 3:**
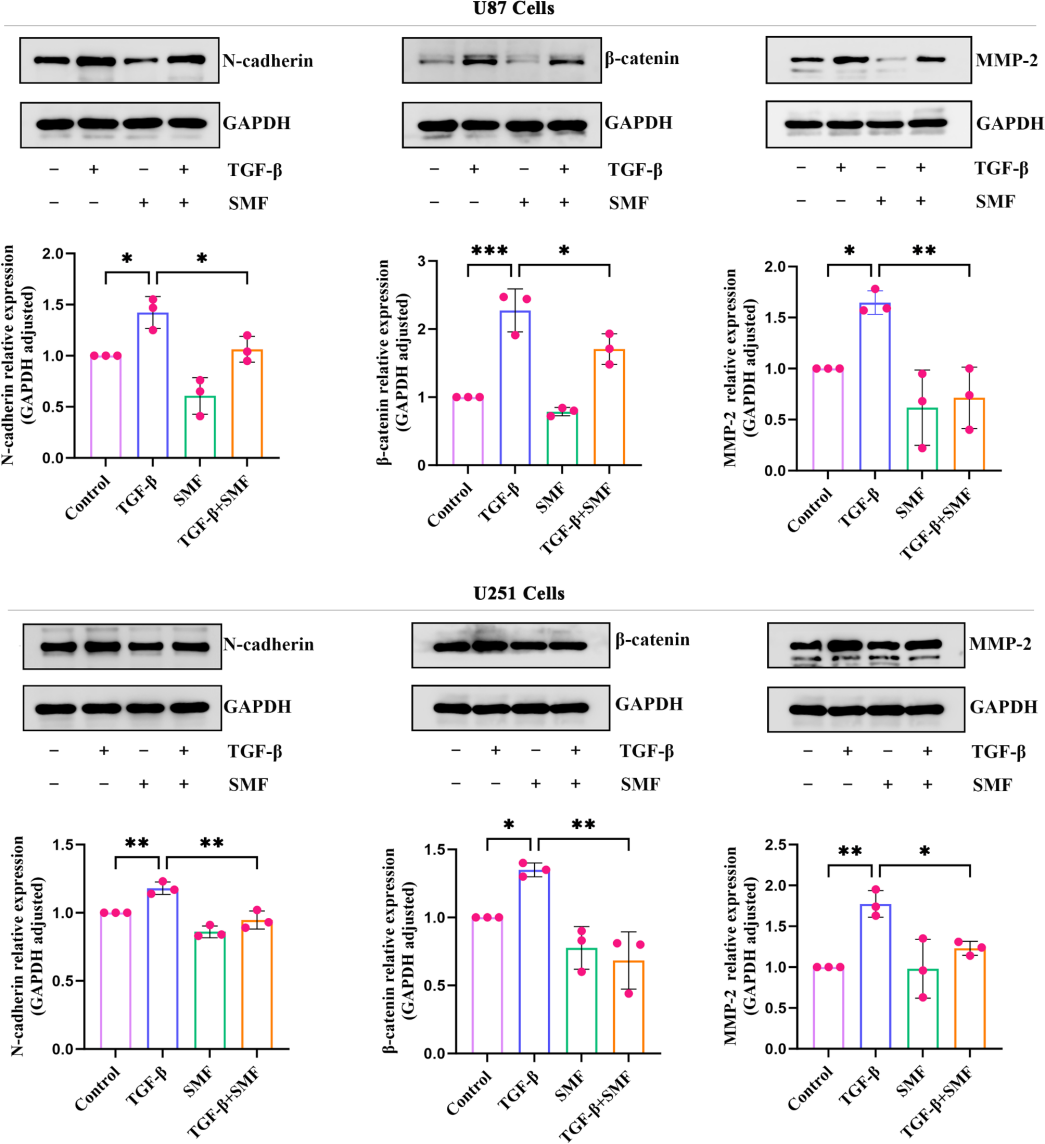
Static magnetic field regulates the expression of EMT-related genes in glioma cells. Expression of EMT-related proteins in U87 and U251 cells under TGF-β1 and static magnetic field intervention. Original blots/gels are presented in Supplementary Information. ns: *p* > 0.05; *p < 0.05; **p < 0.01; ***p < 0.001; ****p < 0.0001.

To confirm whether the static magnetic field regulates the expression of EMT-related genes, we conducted Western blot analysis to assess the protein expression of EMT markers. The results revealed that, compared to the control group, U87 and U251 cells exposed solely to the static magnetic field showed decreased protein levels of mesenchymal markers N-cadherin and β-catenin, as well as reduced expression of matrix metalloproteinase-2 (MMP-2). After 72 h of TGF-β1 treatment (10 ng/ml), both U87 and U251 cells exhibited significantly increased protein expression of N-cadherin, β-catenin, and MMP-2. However, when a static magnetic field was applied in combination with TGF-β1, the co-expression of N-cadherin, β-catenin, and MMP-2 was relatively reduced compared to TGF-β1 treatment alone (Fig. 3). In summary, these results suggest that the static magnetic field can inhibit TGF-β1- induced EMT in glioblastoma cells.

## Discussion

This study investigates the inhibitory effects of a static magnetic field (SMF, 1000 ± 100 Gs) on epithelial-mesenchymal transition (EMT) in glioma cells. Our findings demonstrate that SMF exerts a significant suppressive effect on EMT induced by TGF-β1 in glioma cells, reducing migration and invasion capabilities while notably increasing apoptosis. Through Western blot analysis, we observed that SMF treatment decreased the expression of key EMT markers, including N-cadherin, β-catenin, and MMP-2. These results highlight the potential of SMF as an adjuvant therapy in glioma therapy, offering a novel approach to counteract tumor metastasis.

First, our findings align with previous research showing that TGF-β1, as a primary inducer of EMT, enhances glioma cell migration and invasion^23,24^. TGF-β1 induces morphological changes in cells, promoting a mesenchymal phenotype and increasing cell invasiveness. This is consistent with earlier studies that underscore the pivotal role of TGF-β signaling in advancing malignancy in glioma cells^25^. Although the mechanisms underlying TGF-β-induced EMT have been extensively studied, the process remains complex and highly dependent on the tumor microenvironment^26,27^. As a result, inhibiting TGF-β1-induced EMT is a critical challenge in glioma treatment.

The application of SMF in glioma treatment remains in an exploratory phase. Previous studies have shown that SMF can inhibit cell proliferation and promote apoptosis in certain tumor types^28,29^. Our findings reveal that SMF markedly suppresses TGF-β1-induced EMT, significantly reducing migration and invasion capabilities in glioma cells. Specifically, SMF treatment downregulated N-cadherin and β-catenin expression, markers commonly associated with decreased cell-cell adhesion and increased motility^30^. Additionally, the downregulation of MMP-2 likely plays a role in reducing basement membrane and extracellular matrix degradation^31^, thus restricting the invasive behavior of glioma cells.

The impact of SMF on apoptosis is another significant finding of this study. While apoptosis decreased significantly in TGF-β1-treated glioma cells, the combination of TGF-β1 and SMF notably increased apoptotic rates, suggesting a distinct mechanism by which SMF enhances cell apoptosis. Previous studies suggest that SMF may inhibit glioma cell proliferation by affecting the expression of cell cycle-related proteins^22^. However, our results indicate that SMF’s mechanism of action in promoting apoptosis and suppressing EMT might operate independently of its effects on cell proliferation. Future studies should explore the specific regulatory effects of SMF on apoptotic signaling pathways in glioma cells to further elucidate its therapeutic mechanism.

Furthermore, this study proposes a potential therapeutic strategy utilizing SMF as an inhibitor of TGF-β1-induced EMT. Due to the complexity of the TGF-β signaling pathway^32^, direct inhibition presents certain challenges and potential side effects^33^. SMF, as a non-invasive physical intervention, may effectively reduce glioma cell migration and invasion by downregulating EMT-related markers and promoting apoptosis. However, given that the biological effects of SMF vary depending on experimental conditions such as field strength, exposure duration, and cell type^34^, additional in vivo experiments are essential to validate SMF’s therapeutic efficacy and safety.

In conclusion, this study provides novel insights into the inhibitory role of static magnetic fields in TGF-β1-induced EMT, significantly reducing glioma cell migration and invasiveness. SMF may serve as a unique physical intervention by downregulating EMT-related markers and promoting apoptosis, thus offering a promising adjunct to glioma treatment. Given the variability in SMF effects across different cell types and in vivo conditions, future research should further investigate SMF’s mechanisms in glioma treatment to optimize clinical applications.

## Electronic supplementary material

Below is the link to the electronic supplementary material.

Supplementary Material 1

## Data Availability

The datasets used and/or analysed during the current study available from the corresponding author on reasonable request.
