# Supplementary figures and images for: Static magnetic field inhibits epithelial mesenchymal transition and metastasis of glioma
Source: Sci Rep. 2025 Apr 11;15:12430. doi: 10.1038/s41598-025-96047-x (PMC11992211; doi:10.1038/s41598-025-96047-x)

## Slide 1
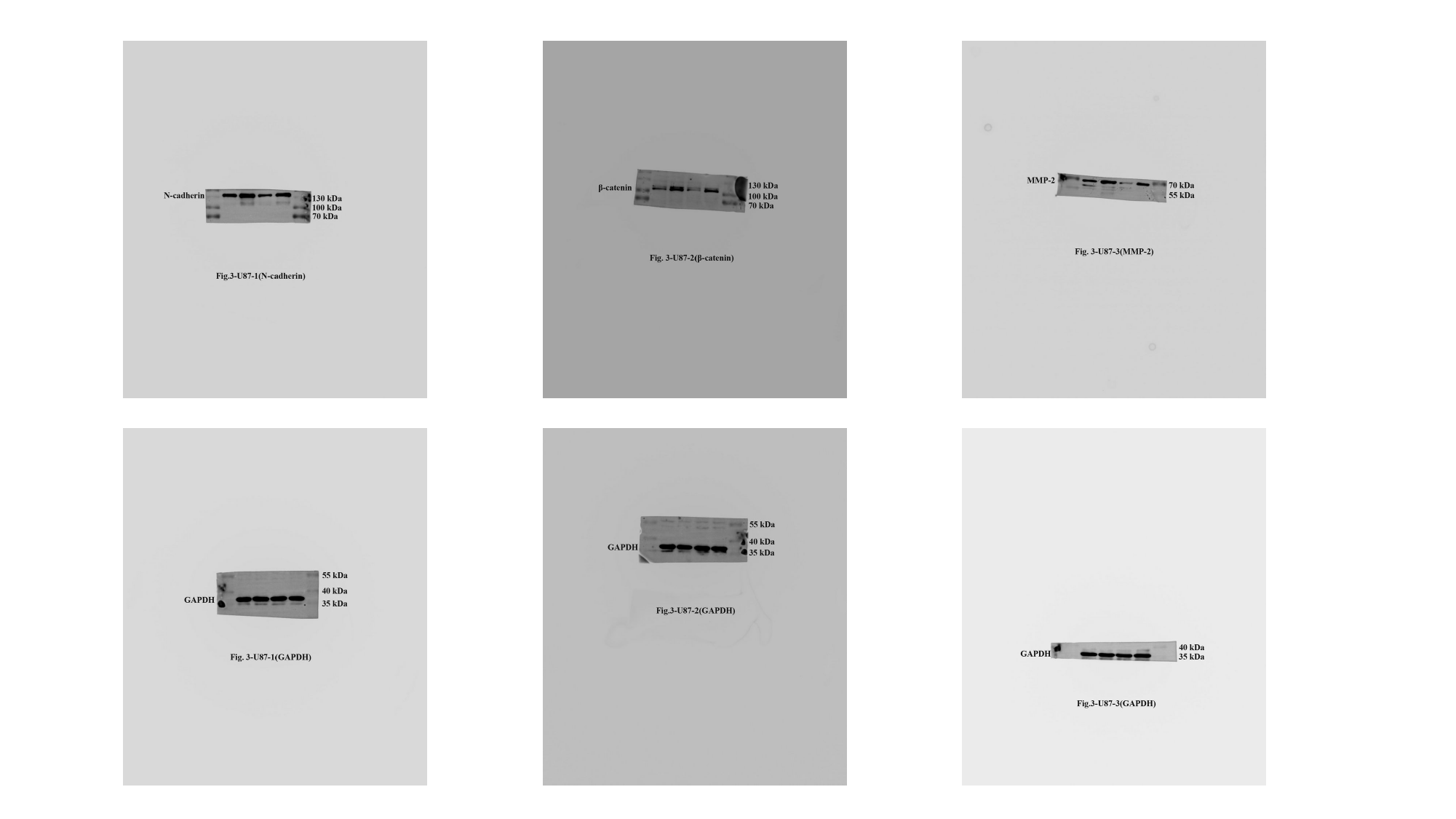

## Slide 2
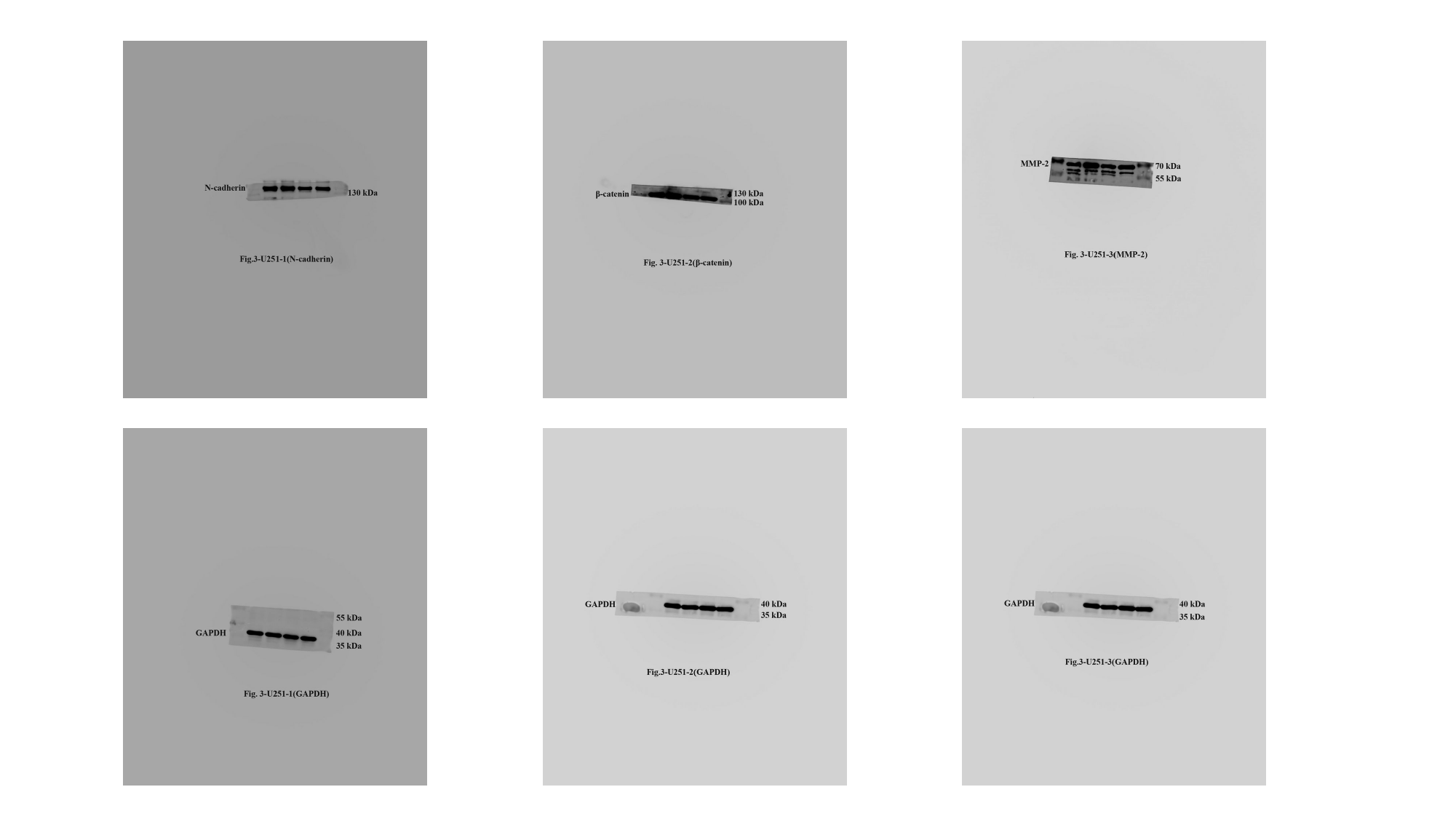

Supplement: Supplementary file 1 — Supplementary Material 1 [file 41598_2025_96047_MOESM1_ESM.pptx]
